# “We All Gonna Get It in Due Time”: Role of Early Life Environmental Racism on Late-Life Functional Ability

**DOI:** 10.1093/geroni/igaf122.546

**Published:** 2025-12-31

**Authors:** Paris Adkins-Jackson, Cailyn Clemons, Muriel Taks Calle, Yoshira Ornelas Van Horne

**Affiliations:** Mailman School of Public Health, Columbia University, New York, New York, United States; Columbia University Mailman School of Public Health, New York, New York, United States; Columbia University Mailman School of Public Health, New York, New York, United States; University of California, Los Angeles, Los Angeles, California, United States

## Abstract

From the National Environmental Policy Act to the Disaster Mitigation Act, the 2000s ushered in a new wave of environmental justice policies meant to protect marginalized groups. Although emergent research on adverse childhood exposures like racism reflects its enduring impact on longevity. This study examines the associations between four measures of environmental racism occurring in early-life and adulthood on late-life functional ability (mobility, independent living, and self-care) among adults racialized as Black (65+) from the Behavioral Risk Factor Surveillance System. We used FEMA, HOLC, IPUMS, and the EPA’s Water Quality Portal, Landfill Methane Outreach, and Superfund data. Exposures included U.S. counties with: (1) a greater number of natural disasters than the national median (2023); (2) fewer water quality monitoring sites than the national median (1960); (3) at least one active landfill site (1990); and (4) at least one Superfund site on the National Priority List (1990). Counties were scored a “1” for Variable 1 if it had a history of redlining (1930-40s), for Variables 2-4, if it had a higher-than-national-average proportion of residents racialized as Black. We averaged county scores to the state-level and performed logistic regressions with functional ability. Living in a state with a greater number of counties with fewer water quality monitoring sites than the national median in areas with greater residents racialized as Black is associated with lower self-care in 2015, and in 2020, lower mobility, independent living, and self-care. Environmental racism during early life disrupts the longevity of residents racialized as Black in later-life.

